# Critical Analysis and Optimization of Stoichiometric Ratio of Drug-Coformer on Cocrystal Design: Molecular Docking, In Vitro and In Vivo Assessment

**DOI:** 10.3390/ph16020284

**Published:** 2023-02-13

**Authors:** Manami Dhibar, Santanu Chakraborty, Souvik Basak, Paramita Pattanayak, Tanmay Chatterjee, Balaram Ghosh, Mohamed Raafat, Mohammed A. S. Abourehab

**Affiliations:** 1 Formulation Development Research Unit, Department of Pharmaceutics, Dr. B. C. Roy College of Pharmacy and Allied Health Sciences, Durgapur 713206, India; 2 Department of Chemistry, Birla Institute of Technology and Science-Pilani, Hyderabad Campus, Shamirpet, Hyderabad 500078, India; 3 Department of Pharmacy, Birla Institute of Technology and Science-Pilani, Hyderabad Campus, Shamirpet, Hyderabad 500078, India; 4 Department of Pharmacology and Toxicology, College of Pharmacy, Umm Al-Qura University, Makkah 21955, Saudi Arabia; 5 Department of Pharmaceutics and Industrial Pharmacy, College of Pharmacy, Minia University, Minia 61519, Egypt

**Keywords:** cocrystal design, molecular docking, stoichiometric ratio, telmisartan, solubility, in-vivo

## Abstract

In this present research, an attempt has been made to address the influence of drug-coformer stoichiometric ratio on cocrystal design and its impact on improvement of solubility and dissolution, as well as bioavailability of poorly soluble telmisartan. The chemistry behind cocrystallization and the optimization of drug-coformer molar ratio were explored by the molecular docking approach, and theoretical were implemented practically to solve the solubility as well as bioavailability related issues of telmisartan. A new multicomponent solid form, i.e., cocrystal, was fabricated using different molar ratios of telmisartan and maleic acid, and characterized by SEM, DSC and XRD studies. The molecular docking study suggested that specific molar ratios of drug-coformer can successfully cluster with each other and form a specific geometry with favourable energy conformation to form cocrystals. Synthesized telmisartan-maleic acid cocrystals showed remarkable improvement in solubility and dissolution of telmisartan by 9.08-fold and 3.11-fold, respectively. A SEM study revealed the formation of cocrystals of telmisartan when treated with maleic acid. DSC and XRD studies also confirmed the conversion of crystalline telmisartan into its cocrystal state upon treating with maleic acid. Preclinical investigation revealed significant improvement in the efficacy of optimized cocrystals in terms of plasma drug concentration, indicating enhanced bioavailability through improved solubility as well as dissolution of telmisartan cocrystals. The present research concluded that molecular docking is an important path in selecting an appropriate stoichiometric ratio of telmisartan: maleic acid to form cocrystals and improve the solubility, dissolution, and bioavailability of poorly soluble telmisartan.

## 1. Introduction

Solubility and dissolution are the key factors for assessing the efficacy of an active pharmaceutical ingredient. Limited solubility of an API is a realistic challenge for the formulation designer to design a suitable dosage form. Among the newly developed chemical entities, more than 70% are poorly water-soluble and highly lipophilic in nature [1,2]. This low solubility may lead to slow dissolution, ineffective absorption, and sub-therapeutical efficacy in patients, as well as low bioavailability [3,4]. Therefore, selecting a suitable method to enhance solubility and dissolution is a crucial role for researchers. Several methods have been reported to improve the solubility and dissolution of poorly water-soluble APIs, such as micronization, salt formation, pH adjustment, incorporation of surfactant, amorphization, complexation, solid dispersion, cocrystals, etc. [5,6,7,8]. Recently, pharmaceutical cocrystals and cocrystallization techniques have been extensively explored by researchers and pharmaceutical industries to improve the solubility, dissolution, and bioavailability of poorly water-soluble drugs. Cocrystals are stoichiometric multi-component new solid forms attached by non-covalent interactions [9,10]. A pharmaceutical cocrystal is a combination of active pharmaceutical ingredients and an appropriate coformer, which are synthesized based on supramolecular chemistry and connected by intermolecular interactions, especially hydrogen bonds and van der Waals forces. Cocrystals have the potential to alter the inherent physicochemical properties of an API, such as solubility, dissolution, stability, etc., without changing the API’s therapeutic efficacy [11,12,13,14].

Telmisartan, a BCS class II drug, is a nonpeptide angiotensin-II receptor antagonist (ARB), indicated for the treatment of hypertension [15,16]. The main drawback of this drug is its very low aqueous solubility, which leads to poor dissolution [17]. These poor solubility and poor dissolution issues limit its oral bioavailability (42–52%) [18]. Several formulation strategies were adopted by the formulation scientists to conquer these issues of telmisartan, such as solid dispersions [19,20,21], nanoparticles [22], incorporations of alkalizers [17], immediate-release tablets [23], amorphous formulations [24], mesoporous nanoparticles [25], nano self-emulsifying drug-delivery systems [26], etc. Apart from the above formulation strategies to improve solubility, the implementation of the crystal engineering approach is a rapidly growing area. There is very little research available on this telmisartan cocrystal design using phthalic acid, citric acid, gallic acid, glutaric acid, and saccharin as coformers [27,28,29,30,31,32]. The telmisartan molecule has one acidic functional group and two basic functional groups, and is suitable for synthon networks (N–H⋯O, N–H⋯N, O–H⋯O and O–H⋯N) for the formation of multicomponent crystals with suitable coformers [33]. However, the chemistry of cocrystallization, its binding dynamics, and the effect of the stoichiometric ratio of drug and carrier towards crystal formation have not been well explored to date [34]. In view of this literature, we aimed to explore a crystal engineering approach to improve the solubility, dissolution, and bioavailability of telmisartan using suitable coformer i.e., maleic acid. 

The objectives of the study may be narrated as the following questions: what is the drug-coformer interaction that may be responsible for cocrystallization [35,36]; what may be the probable stereochemical structures of the drug-coformer complex [37]; and is there any effect of drug-coformer molar ratio during cocrystallization [35,38]? In order to address these problems, we first performed a telmisartan: maleic acid cocrystal dynamic investigation. In this process, the hydrocarbon chain length of the coformer was considered, the thermo-dynamic energy of the drug-coformer complex was elucidated, and the geometry of the subsequent complexes was analyzed. Afterward, the drug:coformer molar ratio was increased continuously, to dissect any downstream effect of it on the geometry and stereochemistry of the complex. Finally, the dipole moment of the complex was estimated, to unravel the crystalline bias of the entire system, assuming the dipole moment to be an index to ascertain favorable geometry and stable ordered binding of the candidate molecules in their minimum energy conformation.

## 2. Results and Discussion

### 2.1. Molecular Docking

The molecular docking was performed in AutoDock Vina and it was observed that the drug-coformer yielded poor binding, probably due to steric repulsion between telmisartan and maleic acid. Also, there being few –OH groups on maleic acid, the formation of hydrogen bonds between maleic acid and telmisartan was not achieved in AutoDock Vina, which may be the reason for the poor binding enthalpy of the two candidates, which could not overwrite the impervious thermodynamic barrier between the two. To address this problem, we applied whole complex energy minimization under MMFF94, using the steepest descent algorithm with steps per update as 100, estimate the energy and most favorable conformation of the complex.

**Figure 1:**
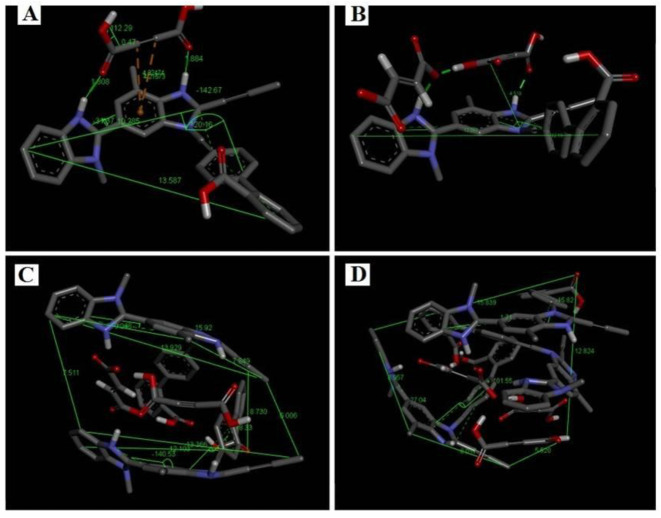
Spatial configuration of TEL-MAL-MALION complex. [((**A**). TEL:MAL:MALION = 1:1:0), ((**B**). TEL:MAL:MALION = 1:1:1), ((**C**). TEL:MAL:MALION = 2:1:1), ((**D**). TEL:MAL:MALION = 3:1:1)].

**Figure 2:**
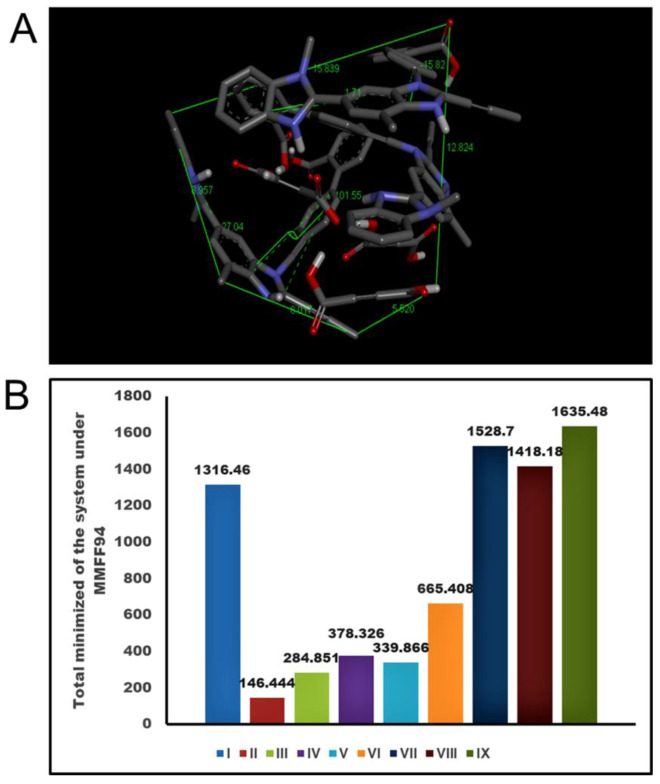
Spatial conformation and energy analysis of TEL-MAL-MALION complex in its most favored arrangement. (**A**). 3D space conformation of TEL:MAL:MALION = 3:1:2, (**B**). Graphical representation of system energy in various molar ratios of TEL-MAL-MALION [(I-Solitary TEL used for docking and complexation); (II-Solitary MAL); (III-Solitary MALION); (IV-TEL:MAL:MALION = 1:1:0); (V-TEL:MAL:MALION = 1:1:1); (VI-TEL:MAL:MALION = 2:1:1); (VII-TEL:MAL:MALION = 3:1:1); (VIII-TEL:MAL:MALION = 3:1:2); (IX-TEL:MAL:MALION = 3:2:1)].

In order to evaluate stability and favorable thermodynamics to form telmisartan-maleic acid cocrystals, molecular-mechanics-guided energy optimization was performed, and the energy of the resulting repertoire was estimated. Telmisartan (TEL) in its docked conformation assumes an energy of 1316.46 KJ/mol; the corresponding energy levels of docked un-ionized maleic acid (MAL) and maleic anion (MALION) individually were found to be 146.444 and 284.851 KJ/mol respectively. Cocrystals with 1:1 molar ratio of telmisartan and maleic acid revealed an energy minimum of 378.326 KJ/mol (Figure 1A), which is well below the consummated energy of individual molecules (1462.904 KJ/mol). Interestingly, when 1 mol of maleic anion (MALION) was doped into this complex (TEL: MAL: MALION of 1:1:1), the energy was further reduced to 339.866 KJ/mol (Figure 1B). The energy measurements were further carried out by varying the TEL or MAL or MALION molar ratio in the cocrystal modeling. For example, a molar ratio of TEL:MAL:MALION of 2:1:1 (Figure 1C) increased the resultant energy of the system to 665.408 KJ/mol, and inserting further hydrophobic TEL (TEL:MAL:MALION of 3:1:1) raised the system energy to 1528.7 KJ/mol (Figure 1D). On the contrary, allowing more MALION into the cocrystal collecting environment (TEL:MAL: MALION of 3:1:2), the energy decreased to 1418.18 KJ/mol (Figure 2A). Further doping with un-ionized MAL (TEL:MAL:MALION of 3:2:1) again elevated the system energy to a maximum of 1635.48 KJ/mol. Thus, it was observed that the molar ratio of telmisartan and maleic acid has a significant impact on the stereochemistry of the cocrystal system. On increasing the molar ratio of telmisartan and maleic acid, the stereochemistry of the system changed, trapping more maleic acid molecules within the system and enhancing the hydrophobic quotient of the system. This further led to buckling away and twisted conformation of telmisartan, probably increasing the stress payload over it. Furthermore, the telmisartan molecules became more buckled, probably due to improved steric interactions between the clusters of molecules, suggesting a further increment of hydrophobicity of the system. This may lead to anti-aggregation and anti-crystalline effects while dissolved in any polar solvent aimed at crystallization. Thus, a strategic amalgamation of the TEL, MAL, and MALION molar ratio governs the system energy during cocrystallization. It subsequently directs the system geometry, bond vectors, bond angles, and torsional angles of accrued cocrystals. The comparative analysis of the system energy with TEL, MAL, and MALION molar ratios is represented in Figure 2B.

### 2.2. Dipole Moment Estimation

**Figure 3:**
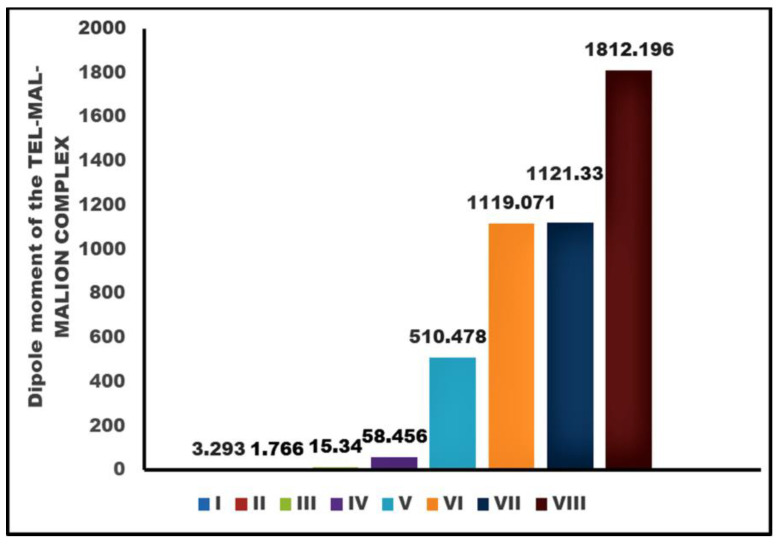
Dipole moment comparison of the co-crystallizing system [(I-Solitary TEL used for docking and complexation); (II-Solitary MAL); (III-Solitary MALION); (IV-TEL:MAL:MALION = 1:1:0); (V-TEL:MAL:MALION = 1:1:1); (VI-TEL:MAL:MALION = 2:1:1);(VII-TEL:MAL:MALION = 3:1:1);(VIII-TEL:MAL:MALION = 3:1:2)].

In order to calibrate the orderly distribution of TEL, MAL, and MALION after strategic molar addition, a dipole moment calculation of the entire system was performed. Since crystal orientation is more symmetric than amorphous, the crystallite repertoire should favor the alignment of poles in an orderly fashion, resulting in a pronounced dipole moment of the system. We found that dipole moments of solitary TEL, MAL, and MALION were 3.293D, 1.766D, and 15.340D respectively. However, the favorable energy conformation of the TEL:MAL (1:1) system increased the dipole moment as high as 58.456D. When we added one mole of MALION into the system (TEL:MAL:MALION = 1:1:1), followed by equilibration through potential energy minimization, the dipole moment reached as high as 510.478D. The introduction of more TEL or MAL or MALION into the system resulted in dipole moments as high as 1119.071 to 1812.196D. The demonstration of dipole moment increment with the increment of molar ratios of the candidates during cocrystallization is illustrated in Figure 3.

### 2.3. Analysis of Conformational Metrics of Drug-Coformer Repertoire

**Figure 4:**
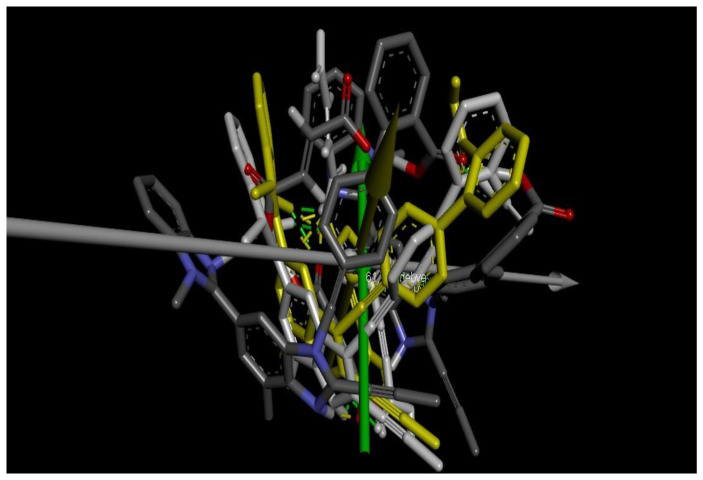
Superimposition of various cocrystallizing systems with increasing molar ratio. [(White-TEL:MAL:MALION = 1:1:1); (Yellow-TEL:MAL:MALION = 2:1:1); (Elemental coloured unit-TEL:MAL:MALION = 3:1:1)].

The conformational metrics and analyses of various stereochemical parameters were performed in Avogadro and Discovery Studio Visualizer. The first interesting point revealed was a change of stereochemistry of the drug and coformer with an increment of molar ratios. For example, when TEL and MAL were introduced in 1:1 molar ratio, the two benzimidazole rings and the terminal aromatic rings showed non-coplanarity, with the torsional angle between the first and second imidazole rings being 31.37° and that between the second imidazole and aromatic ring being 120.16° (Figure 1A). However, when MALION was added into the system (TEL:MAL:MALION; 1:1:1) the corresponding angles changed to 9.58° and 132.19° (Figure 1B). Interestingly, it was also noticed that when a maleate anion was introduced, the system energy was lowered further than its maleic acid counterpart. This is probably due to easier ionic bonding by the maleate anion with the surrounding, stronger, and favorable hydrogen bonding, either with un-ionized maleic acid or with heterocyclic telmisartan, thus helping the system attain more favorable conformation in 3D space. On further addition of TEL with MAL and MALION (2:1:1), such angles became −140.53° (+39°) and −78.33° (+112°) for one telmisartan molecule. The second telmisartan, although, showed a differential bond angle and torsional angle with respect to the first one, when forming box-shaped conformations with the maleates and the first drug molecule (Figure 1C). When further TEL and MALION molecules were introduced (TEL:MAL:MALION; 3:1:2), the stereochemistry of the telmisartan was further changed. For example, the corresponding angles changed to 127.04° and 101.55° (Figure 2A). It may be thus observed that the stereochemistry of the entire cocrystal would vary with strategic alteration of the drug-coformer ratio by gradual deformation of the individual molecular stereochemistry. In order to locate this alteration, we further superimposed the different crystallizing systems’ spatial structures, following the principle of axis-to-axis alignment (Figure 4). Interestingly, it may be noted that the telmisartan backbone from white (TEL:MAL; 1:1) to elemental colored structure (TEL:MAL:MALION; 3:1:2) showed steric repulsion and subsequently bent away from the axis. It may suggest that the increased molar content of the candidate entities leads to an increase of steric or van der Waals repulsion, which is an indirect index of increased hydrophobicity of the cocrystallization system. 

### 2.4. Nature of Bonding Involved in the Cocrystallization

**Figure 5:**
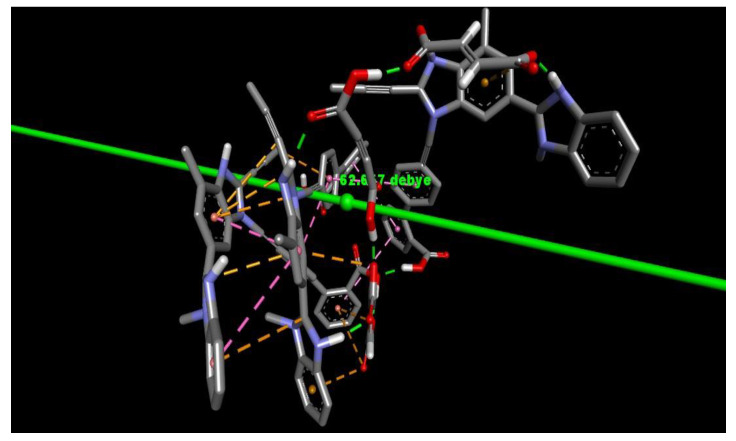
Force of interactions between TEL, MAL and MALION.

The predominant force of interaction between drug and coformer was found to behydrogen bonding. Apart from that, π-π interactions and π-anion interactions are also contributing forces to these interactions (Figure 5). One noteworthy finding is that, for the force of interactions, imidazole rings of the TEL play a major role as acceptors to insert hydrogen bonding with polar carboxylate hydrogens of MAL. 

**Figure 6:**
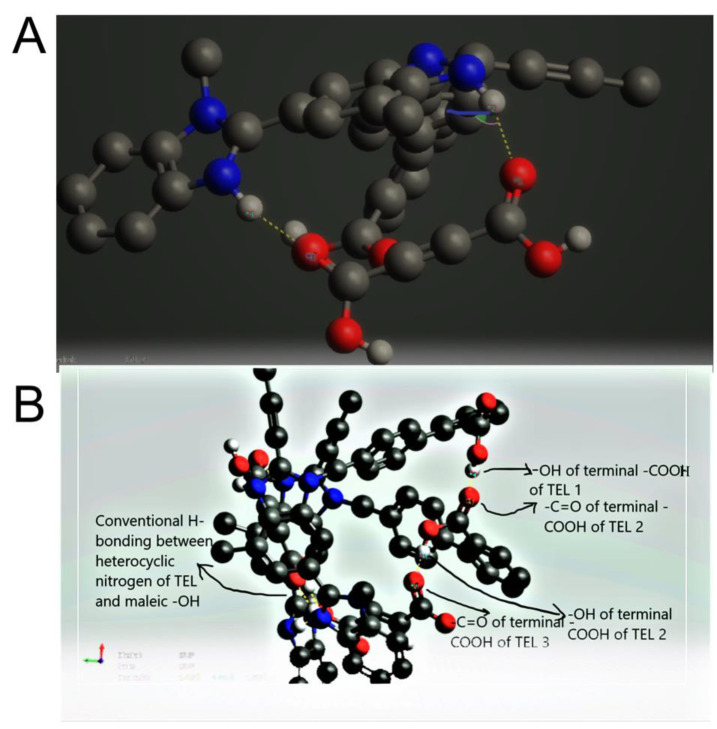
Force of interactions between TEL, MAL and MALION in increasing molar ratio. [((**A**). TEL:MAL:MALION = 1:1:1); ((**B**). TEL:MAL:MALION = 2:1:1)].

Conversely, -–NH of imidazole is revealed to act as a donor, and maleate anions of partially ionized maleic acid are revealed to act as an acceptor. In addition, the polar hydrogens of imidazoles also establish hydrogen bonding interactions with the maleate anion (the electron-dense ionic oxygen terminal). π-π interaction and π-anion interactions are also shown to act as other predominant forces to establish such interactions, which may suggest that drugs containing such aromatic rings or π clouds may contribute more to cocrystallization. Interestingly, in lower molar ratios, these bonds are predominant, where the –COOH group of terminal aromatic rings of TEL are observed vacant in terms of interaction with second TEL or MAL or MALION (Figure 6A). However, when more molecules of MAL or MALIONS are introduced, they reinsert hydrogen bondings even with aromatic polar heads of TEL (Figure 6B). 

The authors suggest that, due to high hydrophobicity and steric interactions, the gradual increase of the molar ratio of telmisartan and maleic acid may lead to a precipitation effect from solvent rather than crystallization. It may also be suggested that, since TEL-MAL 1:1 (un-ionized state) or TEL-MAL-MALION 1:1:1 (ionized state) has the lowest thermodynamic energy amongst all the systems (with increased molar ratio), the unit cell of such a cocrystallization would likely form either of these two systems and its repeated orderly repertoire with polarized terminals (improved dipole moment). The contribution of maleic acid or maleate anions is to concatenate with each other and to reinforce hydrogen bonding onto heterocyclic telmisartan. This finally helps to wrap the latter in one, two, or more dimensions, resulting in the shape of a polygon, which is a hallmark of a cocrystal. 

### 2.5. Equilibrium Solubility Analysis

The solubility study stated that pure telmisartan showed 5.15 ± 1.16 µg/mL solubility in distilled water at 25 °C, whereas, when telmisartan was treated with maleic acid in the presence of solvent and converted into a multicomponent solid form (i.e., cocrystals), a significant (*p* > 0.001) improvement in solubility was observed, and prepared cocrystals were able to enhance solubility by 9.08-fold as compared with the pure drug (Table 1). Improvement in solubility may due to the formation of hydrogen bonds between the imidazole rings of telmisartan and polar carboxylate hydrogens of maleic acid in presence of solvents [39]. 

It was also observed that the molar ratio of drug:coformer has a significant impact on solubility. When the molar ratio of maleic acid was increased from 1:1 to 1:2, the solubility of telmisartan was significantly increased (33.61 ± 2.93 µg/mL to 46.78 ± 3.48 µg/mL). Further, with an increase in the molar ratio of maleic acid in TMA _1:3_, the solubility of telmisartan decreases (i.e., 24.15 ± 2.07 µg/mL) (Table 1). This may be because when the concentration of maleic acid was increased, the hetero-synthon in developed telmisartan-maleic acid cocrystals also increased, which provided substantial strength to the crystal packing. As a result, the breaking of the crystal lattice was also resisted, which resulted in low solubility [31].

From the molecular docking study, it was observed that, on increasing the molar ratio of telmisartan and maleic acid, the stereochemistry of the system changed, entrapping more maleic acid molecules within the system and enhancing the hydrophobic quotient of the system. This further led to buckling away and twisted conformation of telmisartan, probably increasing the stress payload over it. Furthermore, the telmisartan molecules became more buckled, probably due to improved steric interactions between the cluster of molecules, suggesting a further increment of hydrophobicity of the system.

**Figure 7:**
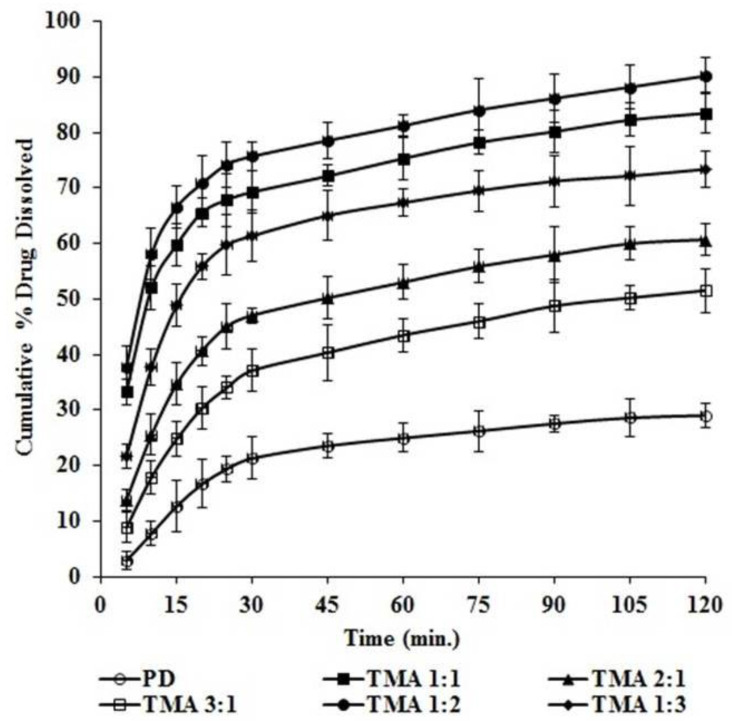
In vitro dissolution study of telmisartan (PD) and telmisartan-maleic acid cocrystals.

The dissolution study was performed in a phosphate buffer (pH 7.5) using a USP dissolution rate test apparatus II (LAB INDIA, DS 8000, Thane, India) at 100 rpm and 37 ± 0.5 °C (Figure 7). This study showed that, for pure telmisartan (PD), 29.04 ± 2.06% drug was dissolved within 120 min. It was also observed that the dissolution of telmisartan was significantly improved by converting it into its cocrystal form. The enhancement in dissolution may be due to the formation of hydrogen bonds between telmisartan and maleic acid in presence of solvents. The computational simulation approach already predicted this finding. Another reason for the enhancement of dissolution may be a reduction in crystal size, solubilization effect of the carrier as well as improved wettability of the drug in presence of solvents [40].

It was also noticed that enhancement in dissolution was significantly dependent on the molar ratio of drug and coformer. When the concentration of telmisartan was gradually increased in the cocrystals, the dissolution decreased significantly. Cocrystal TMA _1:1_ (drug:coformer; 1:1) exhibited a percentage drug dissolution of about 83.46 ± 3.68% within 120 min, cocrystal TMA _2:1_ (drug:coformer; 2:1) exhibited a percentage drug dissolution of about 60.69 ± 2.82% within 120 min, and cocrystal TMA _3:1_ (drug:coformer; 3:1) exhibited only 51.55 ± 3.92% drug dissolution within 120 min (Figure 7). This may suggest that the increased molar content of the drug candidate leads to an increase of steric or van der Waals repulsion, which is an indirect index of increased hydrophobicity of the cocrystallization system, which decreases the solubility as well as dissolution of telmisartan. 

Furthermore, to analyze the impact of the coformer, maleic acid concentration was gradually increased in cocrystal TMA _1:2_ and TMA _1:3_. It was found that with a 1:2 molar ratio of drug:coformer, the dissolution increased markedly, and 90.18 ± 3.17% drug was dissolved within 120 min. However, when the drug:coformer molar ratio was further increased up to 1:3, the dissolution of telmisartan again decreased and only 73.38 ± 3.25% drug was dissolved within 120 min (Figure 7). This may be because when the concentration of maleic acid was increased, the hetero-synthon in developed telmisartan-maleic acid cocrystals was also increased which gives substantial strength to the crystal packing. As a result, the breaking of the crystal lattice is also resisted and results in low solubility as well as less dissolution [31].

The authors suggest that, due to high hydrophobicity and steric interactions, the gradual increase of the molar ratio of telmisartan and maleic acid may lead to a precipitation effect from solvent rather than crystallization. It may also be suggested that since telmisartan:maleic acid 1:1 and 1:2 molar ratio have the lowest thermodynamic energy amongst all the systems (with increased molar ratio), the unit cell of such cocrystallization would likely form of either of these two systems and its repeated orderly repertoire with polarized terminals (improved dipole moment).

From the computational approach prediction data, as well as from the experimental data, it was concluded that the 1:2 molar ratio of drug:coformer (i.e., TMA _1:2_) showed the highest solubility and maximum dissolution i.e., 90.18 ± 3.17% as compared to all other cocrystals, and was selected as an optimized cocrystal batch for further use.

### 2.6. Scanning Electron Microscopic Studies

**Figure 8:**
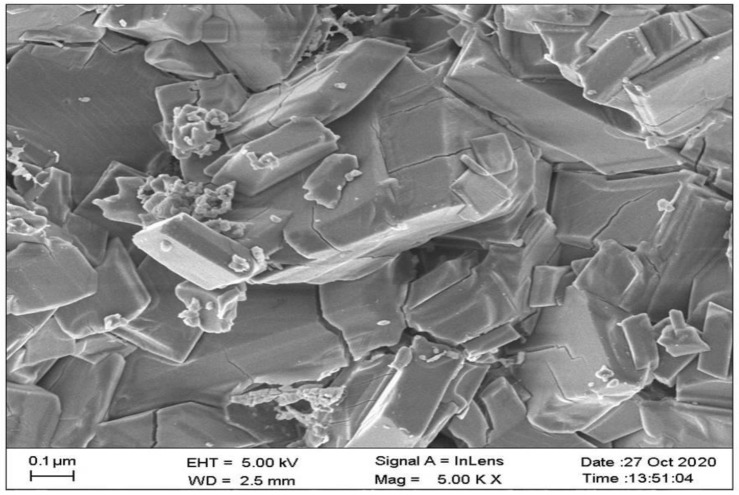
FE-SEM photographs optimized telmisartan-maleic acid cocrystals (TMA _1:2_).

An optimized cocrystal batch (TMA _1:2_) was subjected to FE-SEM study to observe the shape and surface morphology of the prepared cocrystals. This study was performed using a Field Emission Scanning Electron Microscope (FE-SEM, Carl Zeiss, SUPRA 55, Oberkochen, Germany). This study showed that prepared cocrystals have a specific structure with a cubic shape (Figure 8). It was also observed that the surface of the prepared cocrystals was smooth. This study confirmed the formation of a new multi-component solid form i.e., cocrystals.

### 2.7. Differential Scanning Calorimetric Analysis (DSC)

**Figure 9:**
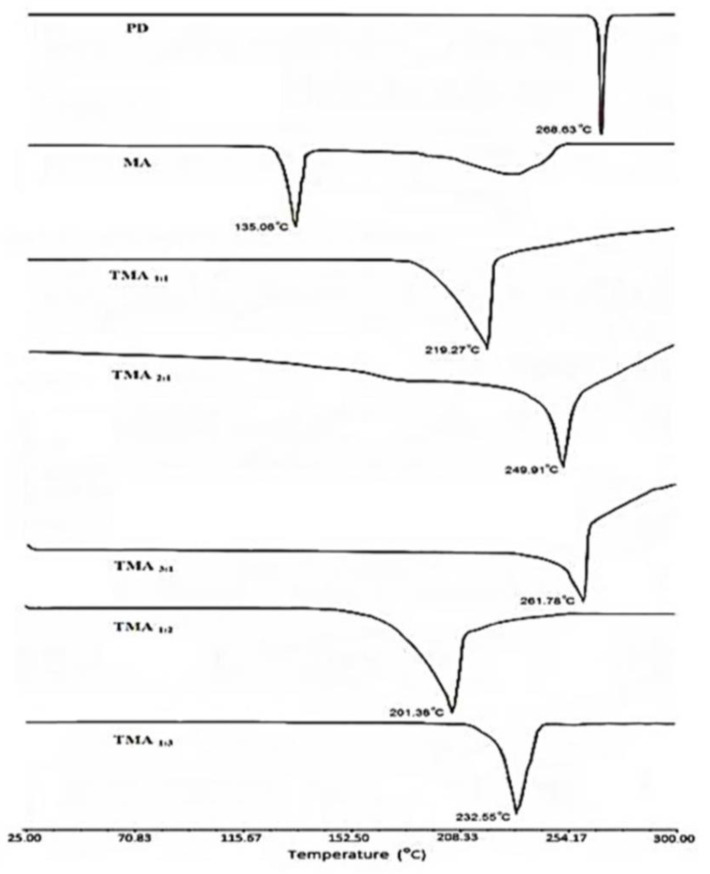
DSC thermogram of telmisartan (PD), maleic acid (MA), and prepared telmisartan-maleic acid cocrystals.

DSC study was performed using a Differential Scanning Calorimeter (Diamond DSC, PYRIS, Perkin Elmer, Shelton, CT, USA) to observe the physicochemical state of samples. This study showed that pure drug (i.e., telmisartan) showed a single melting endothermic peak at 268.63 °C and maleic acid showed a melting endotherm at 135.06 °C (Figure 9). All the prepared cocrystals also showed a single melting endothermic event, which may indicate the existence of a homogenous crystalline phase without any impurity. [31] When the prepared cocrystals were subjected to a DSC study, significant alterations in melting endotherms were observed. It was also noticed that the alterations in melting endotherms was dependent on the molar ratio of telmisartan and maleic acid. Cocrystals with 1:1 molar ratio of telmisartan and maleic acid revealed melting endotherm at 219.27 °C. When the concentration of telmisartan was gradually increased in TMA _2:1_ and TMA _3:1_ cocrystal, melting endothermic events were gradually shifted towards the melting point of pure telmisartan and observed at 249.91 °C and 261.78 °C respectively (Figure 9). This may be because when telmisartan concentration was increased, the telmisartan molecules became more buckled, probably due to improved steric interactions between the clusters of molecules, which increase the hydrophobicity of the system. This may lead to an anti-crystalline effect while dissolved in any polar solvent aimed for cocrystallization. As a result, instead of the formation of a new crystalline phase, a precipitation effect may take place. When the concentration of maleic acid was increased in TMA _1:2_, the melting point of telmisartan was remarkably decreased and observed at 201.38 °C. With further increase in maleic acid concentration in TMA _1:3_, a melting endothermic event was again increased and observed at 232.55 °C (Figure 9). An increase in maleic acid concentration in TMA _1:3_ may give substantial strength to the crystal packing which requires higher energy to break the crystal lattice. Therefore, the DSC study concluded that the melting point of all the prepared cocrystals of different molar ratios was in between the parent molecules (i.e., telmisartan and maleic acid), which may confirm the formation of a new solid phase. 

### 2.8. X-ray Diffraction (XRD) Studies

The X-ray diffraction (XRD) study helped to identify the formation of new solid phases as well as to differentiate new solid phases from the parent compounds based on their different diffraction peaks. The XRD study of telmisartan and prepared cocrystals were studied with an X-Ray Diffractometer (X’Pert Pro, Panalytical, Almelo, The Netherlands). 

**Figure 10:**
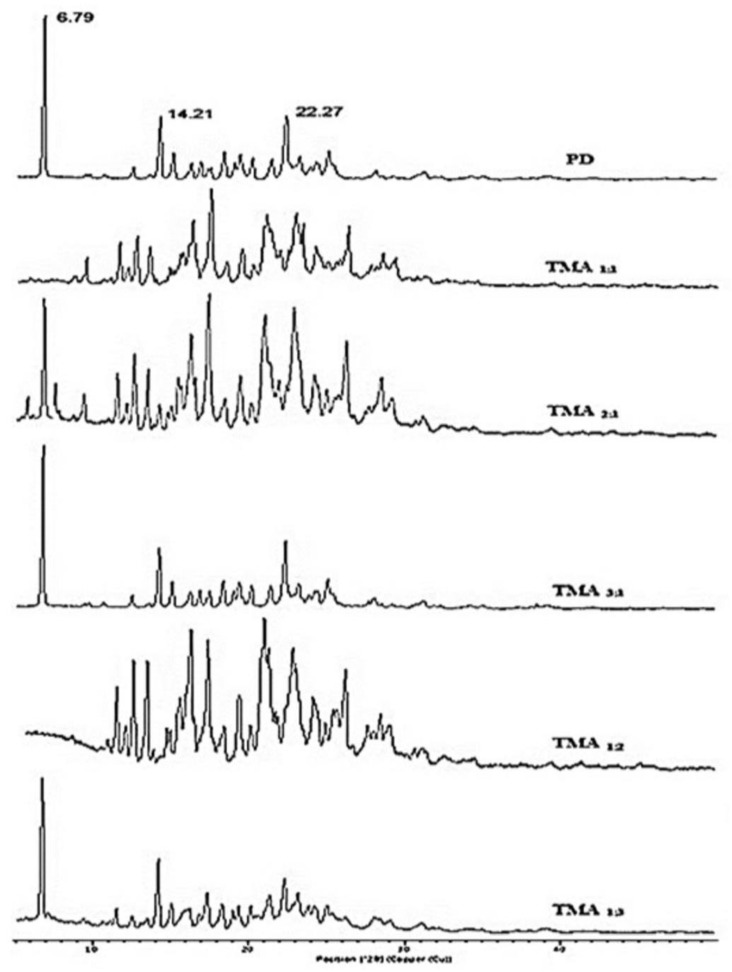
XRD study of telmisartan (PD) and prepared telmisartan-maleic acid cocrystals.

This study revealed that pure telmisartan exhibited different characteristic peaks at 6.79°, 14.21°, 22.27°, etc. among which the 6.79° diffraction peak was the most prominent one (Figure 10). Cocrystals with a molar ratio of 1:1 (drug:coformer; TMA _1:1_) and 1:2 (drug:coformer; TMA _1:2_), showed complete disappearance of the prominent peak of telmisartan (i.e., 6.79°). This finding confirmed the formation of a new solid phase (i.e., cocrystals) with a 1:1 and 1:2 molar ratio of telmisartan and maleic acid. However, in the case of other cocrystals i.e., TMA _2:1_, TMA _3:1,_ and TMA _1:3_ (molar ratio of drug:coformer 2:1, 3:1, and 1:3, respectively), the characteristic peak of telmisartan was unchanged. This observation may reveal that, at a molar ratio of 2:1, 3:1 and 1:3, no prominent new solid phase was not developed. 

**Figure 11:**
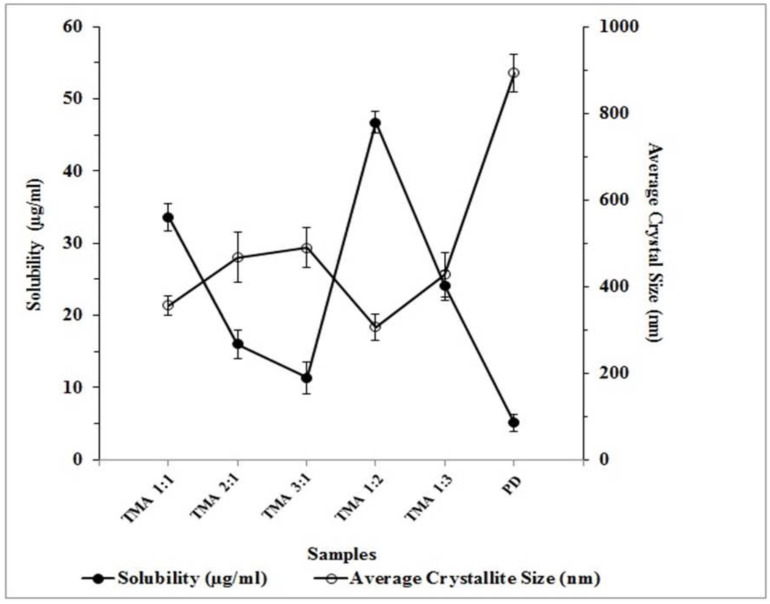
Relationship between crystal size and solubility of telmisartan (PD) and prepared telmisartan-maleic acid cocrystals.

The crystal size of the prepared cocrystals was also determined from PXRD data using Scherrer’s formula. This revealed that, for pure telmisartan, the average crystallite size was 893.51 ± 42.82 nm, whereas the average crystallite size of telmisartan-maleic acid cocrystals was found within the range of 489.86 ± 45.55 nm to 306.10 ± 31.09 nm (Figure 11). It was also interesting to observe a proportional relationship between crystallite size and solubility. When the crystallite size was less, the solubility was found to be greater. The maximum solubility was observed with TMA _1:2_ (i.e., 46.78 ± 3.48 µg/mL), where the crystallite size was lowest (i.e., 306.10 ± 31.09 nm) (Figure 11). Crystallite size also has a significant impact on drug dissolution. When the crystallite size was larger, the dissolution was found to be lesser, whereas, with a smaller crystallite size, the dissolution was greater. Though the size of the crystal was smaller with TMA _1:2_ as compared with other cocrystal batches, the dissolution was found to be the maximum. This may be because a reduction of crystal size helps to increase the effective surface area, allowing more solvent to come in contact with it, as well as improved wettability of the drug in the presence of solvents, which increases the solubility and dissolution of poorly soluble telmisartan [41].

### 2.9. Fourier Transform Infrared Analysis (FTIR)

**Figure 12:**
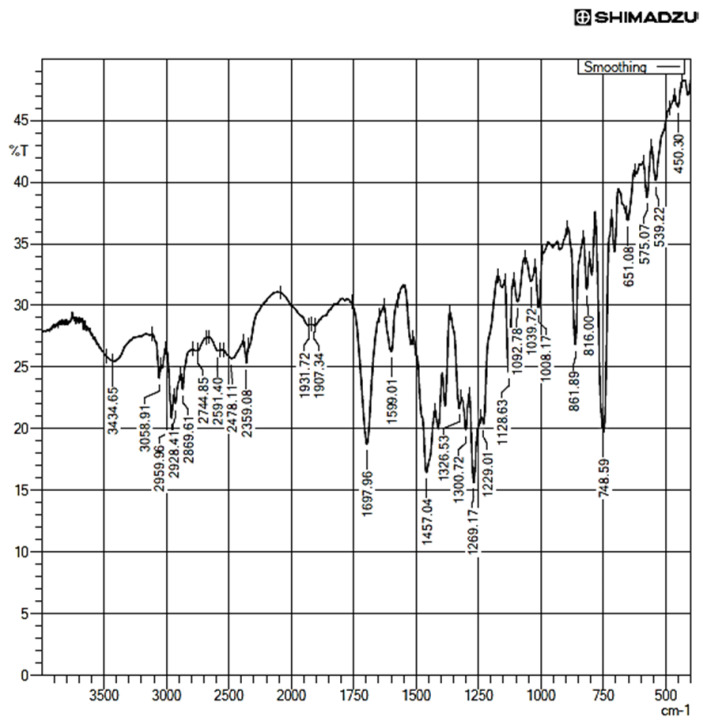
FT-IR spectra of Pure Telmisartan.

The FTIR spectra of the pure drug, i.e., telmisartan, showed different characteristic peaks at 3434.65 cm^−1^, 3058.91 cm^−1^, 2959.96 cm^−1^, 1697.96 cm^−1^, 1599.01 cm^−1^, 1457.04 cm^−1^, 1269.17 cm^−1^, 1128.63 cm^−1^, 1039.72 cm^−1^, and 748.59 cm^−1^ (Figure 12). Among all the peaks, some major peaks are 3434.65 cm^−1^ for -N-H stretch in telmisartan (due to resonance shift of hydrogen in the imidazole rings), 3058.91 cm^−1^ for aromatic C–H stretching, 2959.96 cm^−1^ for aliphatic C-H (-CH_3_) stretching, 2928.21 cm^−1^ for characteristic -CH_2_ stretching, 2869.61 for characteristic N-CH_3_ stretching, 1697.96 cm^−1^ for carbonyl C=O stretching, and 1599.01 cm^−1^ for imine C=N stretching [41]. The peaks at 1229.01 cm^−1^, 1269.17 cm^−1^, 1300.72 cm^−1^, and 1326.53 cm^−1^ depict aromatic -C-N-C- asymmetric stretching for two aromatic nitrogens in two imidazole rings. The peak at 748 cm^−1^ is a characteristic –C=C-– bend of the aromatic rings. 

**Figure 13:**
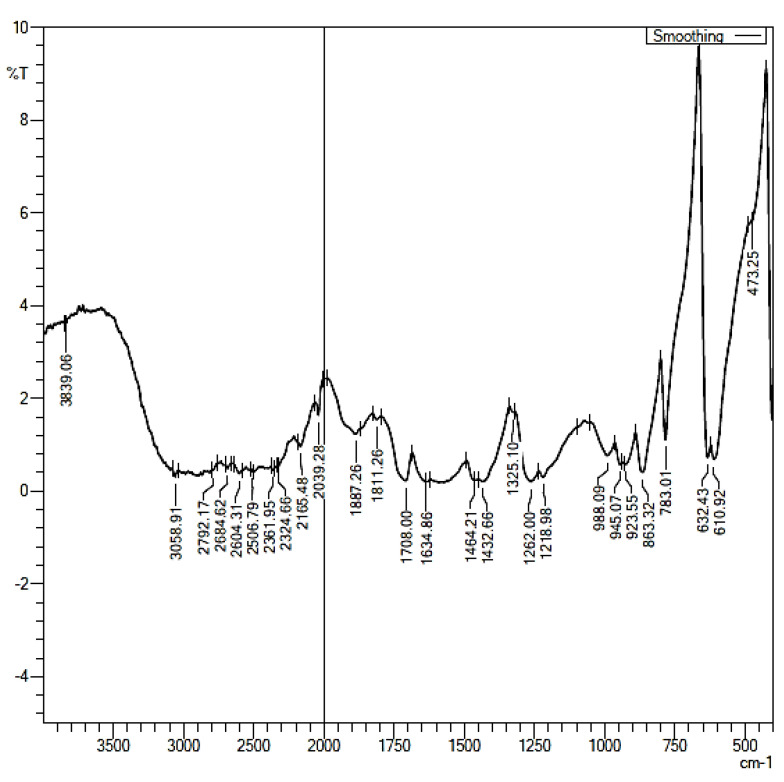
FT-IR Spectra of Pure Maleic acid.

For maleic acid FT-IR spectra (Figure 13), the band at 3058 cm^−1^ depicts the characteristic C-H stretch of –C=C– the backbone of maleic acid; the peak at 1708 cm^−1^ depicts the characteristic C=O stretch of –COOH; and the peaks at 1432.66 cm^−1^ and 1464.21 cm^−1^ suggest the –OH bend of the carboxylic acid groups of maleic acid. The 1262.00 cm^−1^ and 1218.98 cm^−1^ peaks are the characteristic –C=O stretch of two carboxylic groups of maleic acid. The 1708 cm^−1^ and 1620 cm-1 peaks denote the –C=O vibrations of the two carboxylic acids in maleic acid.

**Figure 14:**
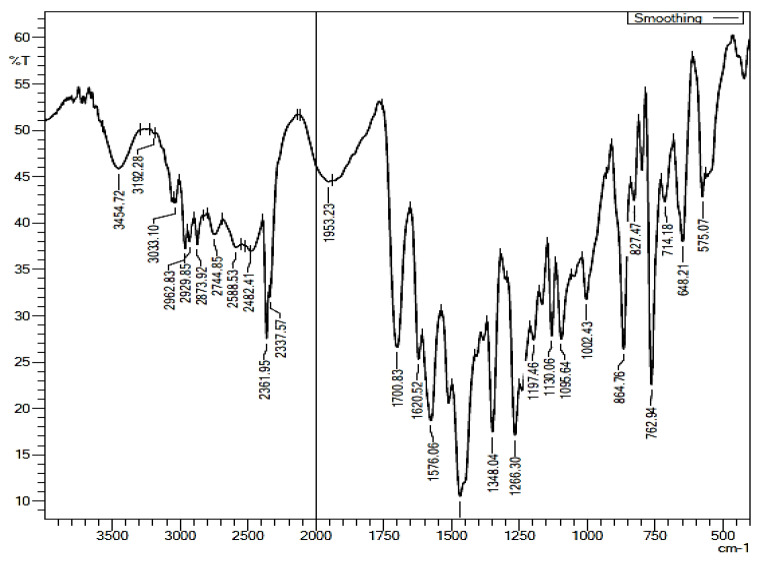
FT-IR Spectra of Telmisartan-Maleic acid Cocrystal (1:1).

It was observed from the FTIR study that the carbonyl C=O stretching peak of pure telmisartan at 1697 cm^−1^ was shifted to 1576.06 cm^−1^ [27] in the optimized cocrystals (Figure 14). This observation revealed the development of supramolecular hetero synthon in the optimized cocrystals [42]. As per the literature, carboxylate ions have a prominent peak from 1540 cm^−1^ to 1650 cm^−1^ [43,44]. When we looked at the peaks, the peak at 3434 cm^−1^ in pure telmisartan showed a shift of 3454 cm^−1^, showing hydrogen bonding involved with the N-H group of telmisartan. However, the N-CH_3_ peak at 2873 cm^−1^ at pure cocrystal showed no shift from pure telmisartan, hence it could be deduced that the N-CH_3_ group of the drug did not participate in any bond formation in the cocrystal. Plus, changes were observed at the aromatic C-N-C peak in the cocrystal (1266.30 cm^−1^ and 1348.04 cm^−1^) compared to that of telmisartan, as stated above; hence, deduction of imidazole participation in hydrogen or π-π bonding could not be omitted. In addition, the aromatic peak of the drug shifted to 3033.10 cm^−1^ in the cocrystal, indicating that there may be some bond interaction, probably π-π overlap with the unsaturated maleic acid. 

Another interesting phenomenon was observed: the broad peaks of maleic acid at 1634.86 cm^−1^ (C=O group of one carboxylate) and two broad C-O stretch peaks of acidic C-OH at 1218.98 cm^−1^ and 1262.00 cm^−1^ were sharpened in the co-crystal, suggesting that the hydrogen bonding between maleic acid dimers have been broken during cocrystallization and newer bonds have been formed. The 1634 cm^−1^ peak of maleic acid also shifted to 1620.52 cm^−1^, reinforcing the assumption of newer bonds forming between the conformer and the drug at the C=O region of the former. A new peak of 1197.46 cm^−1^ formed in the cocrystal, which implies a newer association between the drug and conformer during cocrystallization. No significant interaction was observed through the FT-IR spectrum, which could suggest a chemical reaction between telmisartan and maleic acid. All these results are in agreement with the molecular docking results we obtained before. Therefore, the results confirmed that, in the presence of a solvent system when telmisartan was treated with maleic acid, supramolecular hetero synthon cocrystals of telmisartan were developed. 

### 2.10. ^1^H, ^13^C and 2D-NMR Studies

For analyzing the interaction that has arisen from the orientation of telmisartan (TEL) and maleic acid (MAL) in the 3D chemical space, proton-proton and proton-carbon interaction maps between pure drug and cocrystal were acquiesced by ^1^H, ^13^C and 2D-NMR studies. The NMR interaction maps not only provide hints about the possible interactions for forming such cocrystal, but also illustrated relative 3D-geometry of the drug and conformer for forming such cocrystal. 

The pure drug, TEL derived ^1^H NMR spectrum (see Supplementary Figure S1) and ^13^C NMR spectrum (Figure S2) showed the following peaks. 


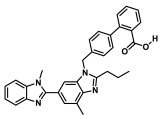


^1^H NMR (400 MHz, CDCl_3_) δ 8.38 (dd, *J* = 8.5, 1.5 Hz, 1H), 8.03 (dd, *J* = 7.6, 1.2 Hz, 1H), 7.47 (m, 2H), 7.41–7.37 (m, 1H), 7.37–7.30 (m, 5H), 7.17 (d, *J* = 8.1 Hz, 2H), 7.06 (dd, *J* = 1.4, 0.8 Hz, 1H), 6.98 (d, *J* = 0.9 Hz, 1H), 5.41 (s, 2H), 3.75 (s, 3H), 3.19–3.07 (m, 2H), 2.70 (s, 3H), 2.00 (dd, *J* = 15.5, 7.6 Hz, 2H), 1.16 (t, *J* = 7.4 Hz, 3H); ^13^C NMR (101 MHz, CDCl_3_) δ 171.11, 156.47, 153.89, 142.76, 141.70, 140.93, 135.49, 134.47, 133.91, 133.62, 130.40, 130.21, 129.33, 128.89, 128.75, 127.39, 127.08, 123.60, 123.19, 121.80, 119.71, 111.31, 109.38, 48.77, 31.77, 29.95, 22.38, 16.95, 14.10 (overlapping peaks are present). LC-MS (EI) calculated for C_33_H_30_N_4_O_2_ [M]^+^: 514.2, found: 514.8.

Similarly, MAL on NMR analysis revealed the following ^1^H NMR spectra (Figure S3).


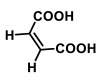


^1^H NMR (400 MHz, DMSO-d_6_) δ 12.71 (s, 2H), 6.26 (s, 2H).

**Figure 15:**
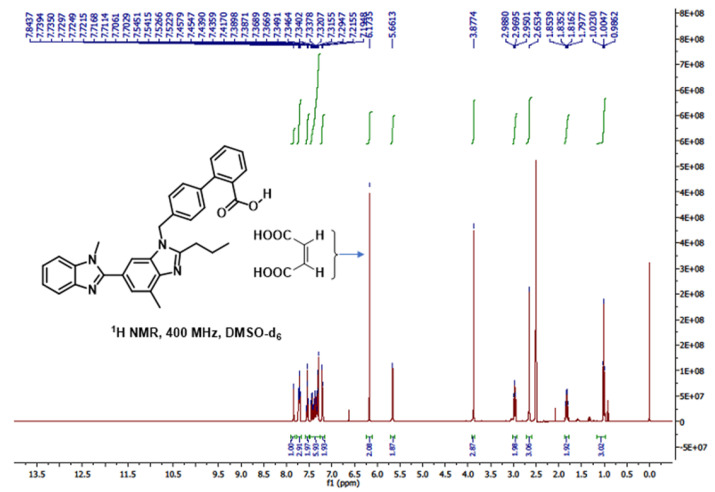
^1^H NMR spectra of TEL-MAL cocrystal.

On acquiring ^1^H NMR spectra of our TEL:MAL cocrystal, it was revealed that the ethylenic proton peak at 6.26 has been shifted to 6.17, which may indicate a change of electromagnetic field of this proton (Figure 15), which we assume arose due to some interaction with TEL. However, no drastic change of peak for either TEL or MAL was observed, thus suggesting that no chemical reaction of covalent bond formation occurred during cocrystallization.

Moreover, the –COOH peak of maleic acid (δ 12.71 (s, 2H)) was totally absent from the cocrystal NMR spectrum. We presume that this is due to the partial ionization of the maleic acid into maleate anion, causing it to lose its acidic proton. Also, the peaks in pure TEL such as 8.38 and 8.03 (likely contributed by the aromatic protons next to –COOH) were shielded in the cocrystal NMR, suggesting the change of electronic field of –COOH after incorporation of the MAL. Thus, it may be inferred that TEL –COOH and MAL –COOH have participated actively in forming the cocrystal. 

**Figure 16:**
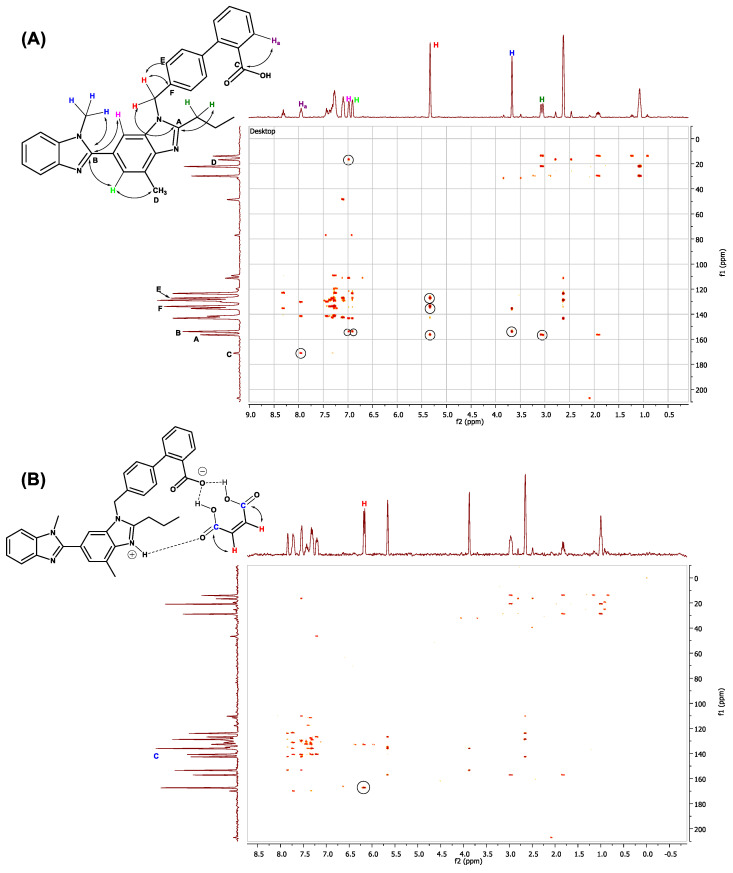
^1^H-^13^C HMBC analysis of (**A**) Pure telmisartan. (**B**) Cocrystal.

In order to study the interactions more in depth in 3D chemical space, we performed 2D ^1^H-^13^C HMBC analysis of the cocrystal (Figure 16).

By looking at the pure TEL HMBC NMR image (Figure 16A) and comparing it with that of the cocrystal (Figure 16B), it may be observed that much of the aromatic proton-carbon interaction has been changed, while the TEL and MAL conjugate has formed cocrystal. A mild-to-moderate peak shift at the aromatic region (δ 7.0–7.5 ppm (^1^H) and δ 120–160 ppm (13C)) suggests a change of ring anisotropy in the presence of MAL. 

**Figure 17:**
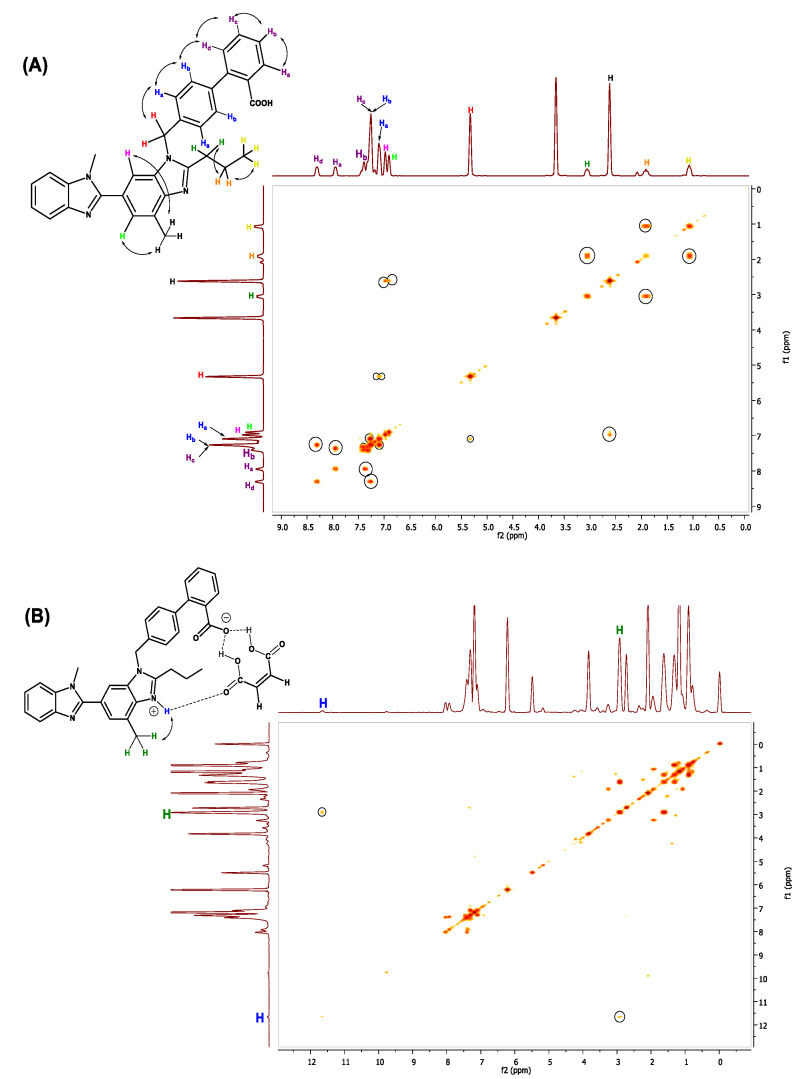
^1^H-^1^H COSY analysis of (**A**) Pure telmisartan. (**B**) Cocrystal.

In order to further confirm this change of chemical shifts and the nature of the interaction, we performed 2D ^1^H COSY analysis of pure TEL (Figure 17A) and of the cocrystal (Figure 17B). A deep interaction was found between the aromatic hydrogen (a downfield shift indicates it is probably imidazolinium and bonded by ionic interaction with the donor nitrogen) as well as the adjacent aryl group. 

**Figure 18:**
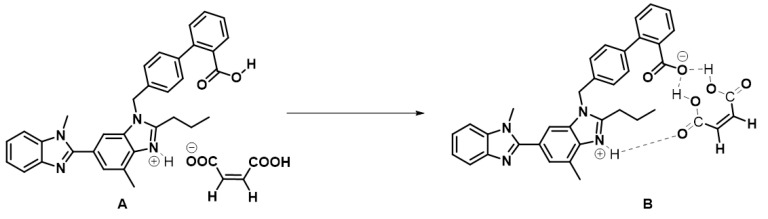
Maleic-acid promoted conversion of (**A**) to (**B**) during the cocrystal formation as hypothesized from the NMR data.

From the integration of the signals of the ^1^H NMR of the cocrystal, it was evident that a 1:1 adduct formed in between the pure drug and the maleic acid. The analysis of the chemical shift values of the pure drug and the cocrystal in the ^1^H NMR revealed that the protons in the aryl ring bearing the –COOH group are shielded by 0.5–0.6 ppm in the cocrystal in comparison to that of the pure drug. This could be only possible if the –COOH group losses its proton. Hence, we speculate that maleic acid would first protonate the most basic N-centre of the drug molecule to form a 1:1 imidazolium maleate salt (Figure 18, structure A) which might undergo another proton exchange process from the carboxylic acid group of the drug to the maleate center (B). Overall, maleic acid is facilitating the pure drug to attain a zwitter ionic form being stabilized by multiple H-bonding in between the zwitter ionic drug molecule and maleic acid (structure B).

The participation of the aromatic ring of telmisartan in the final structure of the cocrystal, the hydrogen bond formation in between –COOH of telmisartan and maleic acid, and the participation of imidazolium N-H to form H-bond interaction with maleic acid –C=O all corroborate the FT-IR and molecular docking data obtained earlier.

### 2.11. Preclinical Studies

**Figure 19:**
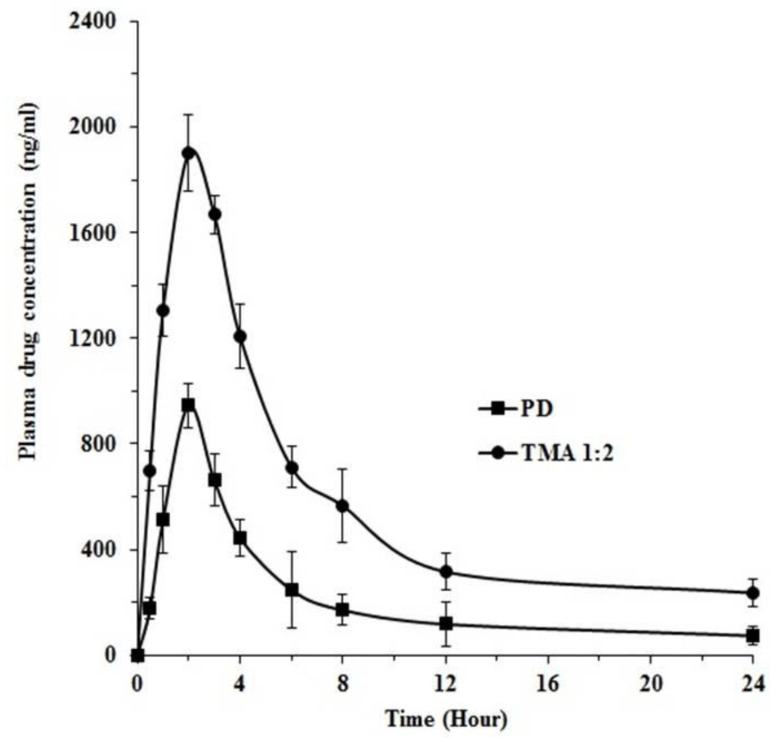
Plasma concentration profile of telmisartan (PD) and optimized telmisartan-maleic acid cocrystals (TMA _1:2_).

This study helps to assess the improvement in oral bioavailability of poorly bioavailable telmisartan after converting it into its cocrystal form. Various pharmacokinetic parameters for pure telmisartan and optimized telmisartan-maleic acid cocrystal (TMA _1:2_) are shown in Table 2 and Figure 19. This study revealed that telmisartan-maleic acid cocrystal (TMA _1:2_) showed a significant increase in peak plasma concentration (C_max_) as well as in area under the curve (AUC_0-∞_) as compared with pure telmisartan. The C_max_ of pure telmisartan and telmisartan-maleic acid cocrystal (TMA _1:2_) were found to be 945.31 ± 27.92 ng/mL and 1900.43 ± 56.33 ng/mL, respectively, whereas the AUC_0-∞_ of telmisartan-maleic acid cocrystal (TMA _1:2_) was found to be increased by 2.63-fold over telmisartan Table 2). The increase in C_max_ with cocrystal formulation can be ascribed to the increase in dissolution as well as the absorption rate of pure telmisartan. The higher plasma drug concentration and higher AUC_0-∞_ of telmisartan-maleic acid cocrystal also signify better solubility and enhanced in vivo absorption as compared with pure telmisartan.

## 3. Materials and Methods

### 3.1. Materials

Telmisartan was supplied by Macleods Pvt. Ltd., Gangtok, India, as a gift sample. Maleic acid and ethanol were procured from Merck, India. All other materials and solvents used were of analytical grade. Molecular structures were constructed in Chem Draw Ultra (Professional, version 15.0, ChembridgeSoft Corporation, PerkinElmer Inc., Waltham, MA, USA), and Minimization of Energy was performed in Avogadro (1.2.0, University of Pittsburgh, Pittsburgh, PA, USA). The energy-minimized structures were processed in MGL tools 1.5.6 (The Scripps Research Institute, La Jolla, CA, USA) and docked in AutoDock Vina (1.1.2, The Scripps Research Institute, La Jolla, CA, USA). The docked structures were further processed in Discovery Studio Visualizer (Dassault Systems, BIOVIA, San Diego, CA, USA) and UCSF Chimera (version 1.13.1, Resource for Biocomputing, Visualization, and Informatics (RBVI), University of California, San Francisco, CA, USA). For determining the stereochemistry, bond lengths, bond angles, and dihedral angles of the candidates during cocrystallization, Avogadro and Discovery Studio Visualizer (version 4.0) were used.

### 3.2. Methods

#### 3.2.1. Computational Simulation Study

A computational simulation was performed to determine the solubility and hybridization of cocrystals, and hence insinuate the probable 3D structure of the cocrystal. The hydrophobicity, steric hindrance, and other such forces of interaction affecting 3D conformation of cocrystals were estimated, and were further correlated with solubility and crystallite repertoires derived on an experimental basis. The docking generated poses of drug-coformer complexes (1:1) were suitably doped with an excess of drug and coformers in increased molar ratios. The subsequently generated drug-coformers were re-subjected to global energy minimization by subjecting the hybrids to Molecular Mechanics simulation. Molecular Mechanics Force Field 94 (MMFF94) was attributed to the molecules, the steepest descent algorithm was undertaken for finding the energy-minimized conformation of the complex, and steps per update were assigned as 100. The drug was placed within a hypothetical grid box with 2 Å spacing, where each point of the grid box depicted the force-field potential of the molecule. The docked generated conformation of the coformer was used as a probe and was manually placed at each corner of the grid point to measure force-field interaction with the drug molecule. The lowest interactive pose indicated by the lowest system energy demonstration under MMFF94 was chosen as the starting point of further system energy minimization. Steepest Descent Algorithm was used, and all the bonds of the molecules were kept rotatable. The complexes with suitable molar ratios thus led to probable stable structures of cocrystals. The hydrophobicity, dipole moment, complex diameter, and other stereochemical descriptors were calculated in Avogadro and Discovery Studio Visualizer. 

#### 3.2.2. Preparation of Structures

The 2D structures of drug and coformer were first prepared in Chem Draw Ultra and the outputs were saved in MDL MOL format. Further, the outputs were re-unlocked in Avogadro and subjected to energy minimization. Molecular Mechanics Force Field 94 (MMFF94) were attributed over the molecules and system energy was minimized by the Steepest Descent algorithm (Steps per update, 100) to obtain their minimum energy conformation. The energy-minimized outputs were saved in PDB format and used for docking.

#### 3.2.3. Processing of Structures for Molecular Docking 

The PDB format of molecular structures was opened in MGL Tools 1.5.6, added with polar hydrogen, and added with both Gasteiger and Kollman charges. Maleic acid was treated as a ligand here, whose bonds were all set free for rotation and torsion. 

#### 3.2.4. Molecular Docking

The docking of telmisartan (drug) and maleic acid (coformer) was performed in AutoDock Vina using standard Monte Carlo algorithms [45]. Dry-state docking was performed to avoid any interference of water molecules or other solvents during docking. Thus, in this stage of docking, a non-ionic form of maleic acid was undertaken, where the ionic state of the same was taken and docked in Avogadro and UCSF Chimera. Telmisartan was caged in a Grid Box of dimension 50 Å × 40 Å × 40 Å with the centers of the x, y, and z coordinates set at 6.847, −2.774, and −0.147. 

Since maleic acid is a weak acid and may partially ionize in these solvents, the ionized form of the maleic acid was also taken in a separate docking, and subsequent bonding interactions were monitored in UCSF Chimera. For studying the interaction of the maleate anion, the anionic structure was further prepared in Chem Draw Ultra, the energy minimized in Avogadro and saved in .mol2 format, and subsequently docked on the maleic-telmisartan system. For tracking the cocrystal formation at the seeding stage, the telmisartan and maleic acid were taken at different molar ratios, where 50% of maleic acid was assumed to be ionized, since it is a weak acid.

#### 3.2.5. Preparation of Telmisartan Cocrystals Using Maleic Acid

Telmisartan-maleic acid cocrystals were prepared using different stoichiometric amounts of telmisartan and maleic acid (Table 1). The molar ratios of the pure drug as well as the coformer were varied to observe the impact of drug:coformer ratio on crystallization. The specific molar ratio of telmisartan (514 mg, 1 mmoL) and maleic acid (116 mg, 1 mmoL) was mixed physically using an ointment slab. Then the drug-coformer powder blend was slowly added to a solvent mixture (i.e., distilled water: ethanol; 1:1) and stirred continuously, using a magnetic stirrer until the ethanol was evaporated out. Then it was transferred into a petri dish and kept at 50 °C for complete drying. Finally, the formed crystalline material was scrapped and stored in a desiccator for further use.

#### 3.2.6. Characterization of Pure Telmisartan and Telmisartan-Maleic Acid Cocrystal

##### Equilibrium Solubility Analysis

The solubility analysis of telmisartan and prepared telmisartan-maleic acid cocrystals were assessed by placing an excessive amount of sample in 50 mL of distilled water at 25 °C. Then it was stirred for 24 h using a magnetic stirrer to ensure that the solution must reach equilibrium, and then sonicated (Imeco Sonifier, Imeco Ultrasonics, Pune, India) for 15 min. Then the solution was filtered and subjected to spectrophotometric analysis at 296 nm, using a UV-visible spectrophotometer (UV-1800 Shimadzu, Kyoto, Japan). Each determination was made in triplicate, and the data are shown in Table 1.

##### Dissolution Studies

A USP dissolution rate test apparatus II (LAB INDIA, DS 8000, India) was used to perform the dissolution studies of pure telmisartan and prepared multicomponent solid form (telmisartan cocrystals) in phosphate buffer medium (pH 7.5) [46] at 37 ± 0.5 °C. An accurately weighed amount of sample was placed in a 900 mL dissolution medium and stirred at 100 rpm. At each specific time interval, a 5 mL sample was withdrawn and replaced by an equal volume of fresh pre-warmed phosphate buffer (pH 7.5) to maintain the sink condition throughout the experiment. The samples were filtered through Whatman filter paper (0.45 µm) and analyzed at 296 nm using a UV-visible spectrophotometer (UV-1800 Shimadzu, Japan). The dissolution studies were conducted in triplicate.

##### Scanning Electron Microscopic Studies

The shape and surface morphology of optimized telmisartan-maleic acid cocrystals were assessed with a Field Emission Scanning Electron Microscope (FE-SEM, Carl Zeiss, SUPRA 55, Oberkochen, Germany) at an acceleration voltage of 5 kV and a chamber pressure of 0.6 mm Hg. The images were taken using a field emission scanning electron microscope.

##### Differential Scanning Calorimetry (DSC) Analysis

Differential scanning calorimetry (DSC) analysis of samples was performed using a Differential Scanning Calorimeter (Diamond DSC, PYRIS, Perkin Elmer, Shelton, USA). The samples were hermetically sealed in perforated aluminium pans and heated at a constant rate of 10 °C/min over a temperature range of 25 °C to 300 °C. The system was purged with nitrogen gas to maintain an inert atmosphere.

##### X-ray Powder Diffraction (XRD) Studies

An XRD study was performed to assess the physico-chemical state of telmisartan in prepared telmisartan-maleic acid cocrystals. The XRD study was performed by X-Ray Diffractometer (X’Pert Pro, Panalytical, Almelo, The Netherlands) using monochromatized Cu Kα radiation (λ = 1.54 Å) at a voltage of 45 kV and a current of 40 mA. Measurements were carried out in the angular scan range from 5° to 50° (2θ) at a scan speed of 1°/min. This study also helps to determine the size of the formed crystallite using Scherrer’s formula.

$$\mathrm{Crystallite}\;\mathrm{size}\;\left(D_{p}\right)=\frac{K\;\lambda }{\left(B\cos\theta \right)},$$

where D_p_ is average crystallite size (nm), K is Scherrer constant, λ is X-ray wavelength, B is FWHM (Full Width at Half Maximum) of XRD peak, and θ is XRD peak position.

##### Fourier Transform Infrared (FTIR) Spectroscopy

FTIR study was recorded on a FTIR analyzer (IR Spirit 00382, Shimadzu, Japan). The dry powder sample was mixed with KBr and pressed into pellets using a KBr pellet press (Kimaya Engineers, Thane, India) at 5 tons pressure and scanned over the range of 4000–450 cm^−1^.

##### 1D and 2D NMR Studies

Nuclear Magnetic Resonance (NMR) spectra (1D and 2D) were recorded on a Bruker 400 MHz (400 MHz for ^1^H NMR and 100 MHz for ^13^C NMR) NMR Spectrometer instrument (Model: Advance Neo, Magnet System: Ascend and Magnet Operation Field: 9.4 Tesla). The analytical data of the ^1^H NMR and ^13^C NMR are reported in parts per million (ppm). The data were measured relative to residual chloroform (7.26 ppm for ^1^H NMR and 77.00 ppm for ^13^C NMR) in the deuterated solvent (CDCl_3_) for the pure drug. The analytical data of the ^13^C NMR spectra were obtained with ^1^H decoupling. Since maleic acid and the mixture of pure drug and maleic acid were sparingly soluble in CDCl_3_, DMSO-D_6_ was used as the solvent and the analytical data were measured relative to residual DMSO (2.5 ppm for ^1^H NMR). Coupling constants were reported in Hz.

### 3.3. Preclinical Studies (In Vivo Studies)

#### 3.3.1. Animals

In vivo study protocol was approved by the Institutional Animal Ethical Committee, Dr. B. C. Roy College of Pharmacy & AHS., Durgapur, West Bengal, India (Approval No: BCRCP/IAEC/8/2019). The pharmacokinetic study was performed using male Wistar rats (180–200 g). The animals were allowed to be acclimatized for a period of 1 week in our laboratory environment prior to the experiment and had free access to water and food. 

#### 3.3.2. Pharmacokinetic Studies in Rats

The animals were divided into two groups. Group 1 (6 animals) received pure telmisartan (1 mg/kg) with 0.5% *w*/*v* sodium carboxymethylcellulose, and Group 2 (6 animals) received optimized cocrystals (equivalent to 1 mg/kg pure telmisartan) with 0.5% *w*/*v* sodium carboxymethylcellulose. Blood samples were collected at predetermined time intervals from the retro-orbital venous plexus of the rats and centrifuged for 5 min at 10,000 rpm. The plasma was separated and drug analysis was carried out by RP-HPLC method [47] using the software ‘Class-Vp series version 5.03 (Shimadzu)’. The in vivo pharmacokinetic parameters such as peak plasma concentration (C_max_), total area under the plasma concentration-time curve (AUC_0–∞_), elimination rate constant (KE), and relative bioavailability (%) were calculated by using Kinetica software (version 4.4.1; Thermo Fisher Scientific, Waltham, MA, USA).

## 4. Conclusions

The impact of molecular docking on cocrystal design was successfully studied in the above research. From the above research, it was concluded that drug-coformer molar ratio had a significant influence on cocrystallization, as well as on the stereochemical structures of the drug-coformer complex. It was also concluded that synthesized telmisartan-maleic acid cocrystals markedly improved the solubility as well as dissolution of poorly soluble telmisartan. In vivo study revealed that prepared cocrystals significantly increased the bioavailability of telmisartan. Finally, it was concluded that molecular docking was an important path to predict and select the appropriate molar ratio of telmisartan-maleic acid that could form cocrystals and improve the solubility, dissolution, and bioavailability of poorly soluble telmisartan.

## Figures and Tables

**Table 1:** Formulation and physico-chemical characterization of telmisartan-maleic acid cocrystals.

Formulation	(Drug:Coformer) (Molar Ratio)	Telmisartan (mg)	Maleic Acid (mg)	Solubility (µg/mL; at 25 °C)
**TMA _1:1_**	1:1	514	116	33.61 ± 1.93
**TMA _2:1_**	2:1	1028	116	16.01 ± 1.99
**TMA _3:1_**	3:1	1542	116	11.38 ± 2.23
**TMA _1:2_**	1:2	514	232	46.78 ± 1.48
**TMA _1:3_**	1:3	514	348	24.15 ± 2.07
**PD**	-	-	-	5.15 ± 1.16

TMA: Telmisartan-maleic acid cocrystals; PD: Pure drug (i.e., Telmisartan). Mean ± SD, n = 3.2.6. Dissolution Studies.

**Table 2:** Comparative pharmacokinetic data of pure telmisartan (PD) and optimized telmisartan-maleic acid cocrystal (TMA _1:2_).

Pharmacokinetic Parameters	PD	TMA _1:2_
**C_max_ (ng/mL)**	945.31 ± 27.92	1900.43 ± 56.33
**AUC_0-∞_ (ng/mL × hr)**	4184.17 ± 87.82	10,956.32 ± 103.27
**K_E_ (h^−1^)**	0.108 ± 0.036	0.184 ± 0.019
**Relative bioavailability**	-	2.62

Mean ± SD., *n* = 6.

## Data Availability

Data is available within the article and Supplementary Materials.

## Supplementary Materials

The following supporting information can be downloaded at: https://www.mdpi.com/article/10.3390/ph16020284/s1, Figure S1: _1_H^1^ NMR spectra of Telmisartan; Figure S2: ^13^C NMR spectra of Telmisartan; Figure S3: _1_H^1^ NMR spectra of maleic acid.
