# Supplementary material for: Critical Analysis and Optimization of Stoichiometric Ratio of Drug-Coformer on Cocrystal Design: Molecular Docking, In Vitro and In Vivo Assessment
Source: Pharmaceuticals (Basel). 2023 Feb 13;16(2):284. doi: 10.3390/ph16020284 (PMC9959501; doi:10.3390/ph16020284)
Supplement: Supplementary file 1 [file pharmaceuticals-16-00284-s001.zip › pharmaceuticals-2004180-supplementary.pdf]

Supplementary Information

Article

Critical Analysis and Optimization of Stoichiometric Ratio of Drug-Coformer on Cocrystal Design: Molecular Docking, *In-Vitro* and *In-Vivo* Assessment

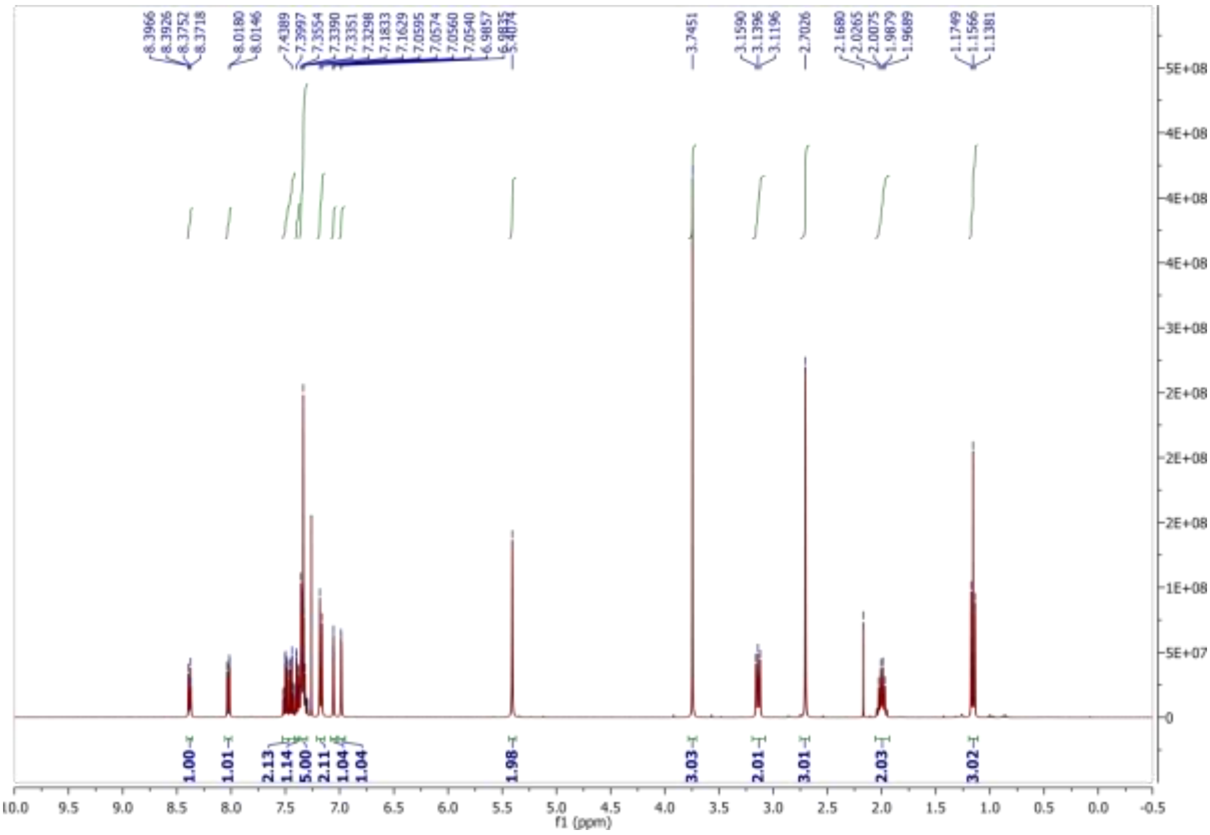

Figure S1. $_{1}H^{1}$ NMR spectra of Telmisartan.

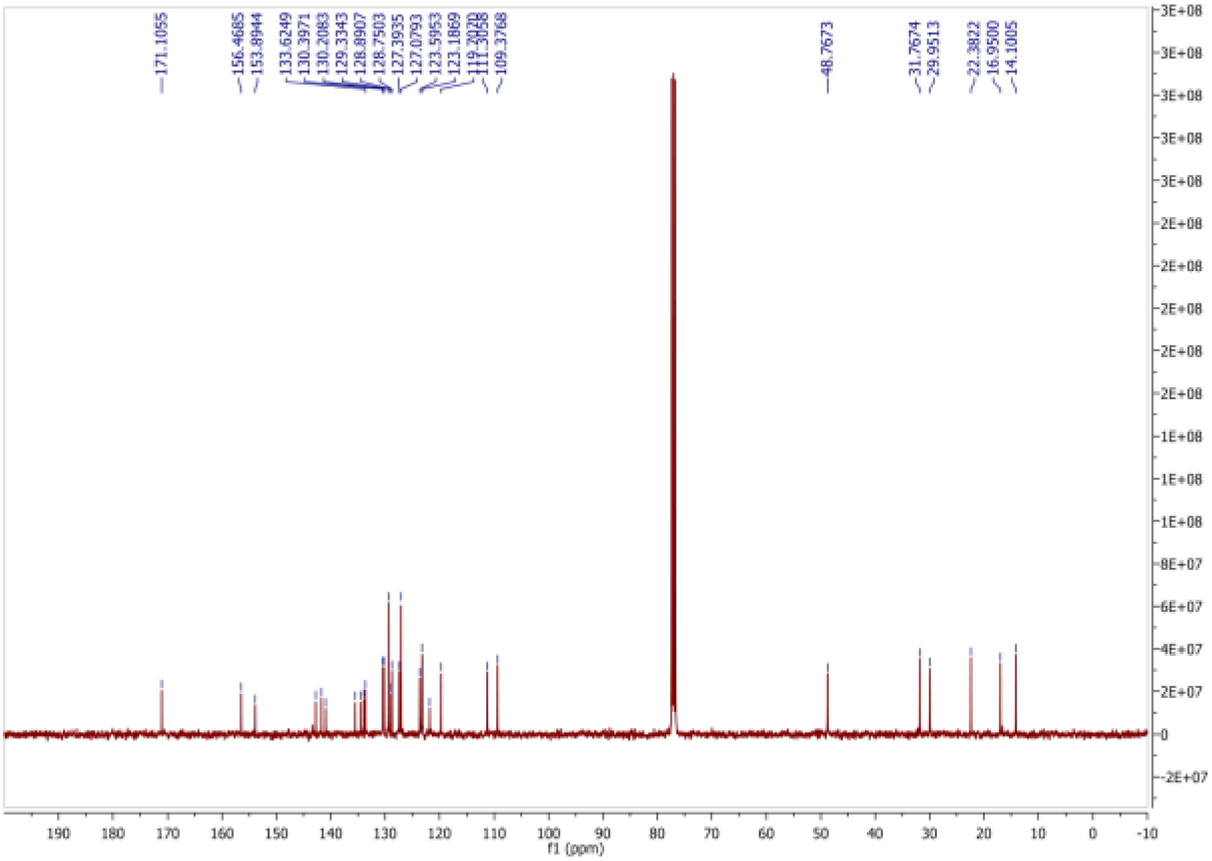

Figure S2. ^{13}C NMR spectra of Telmisartan.

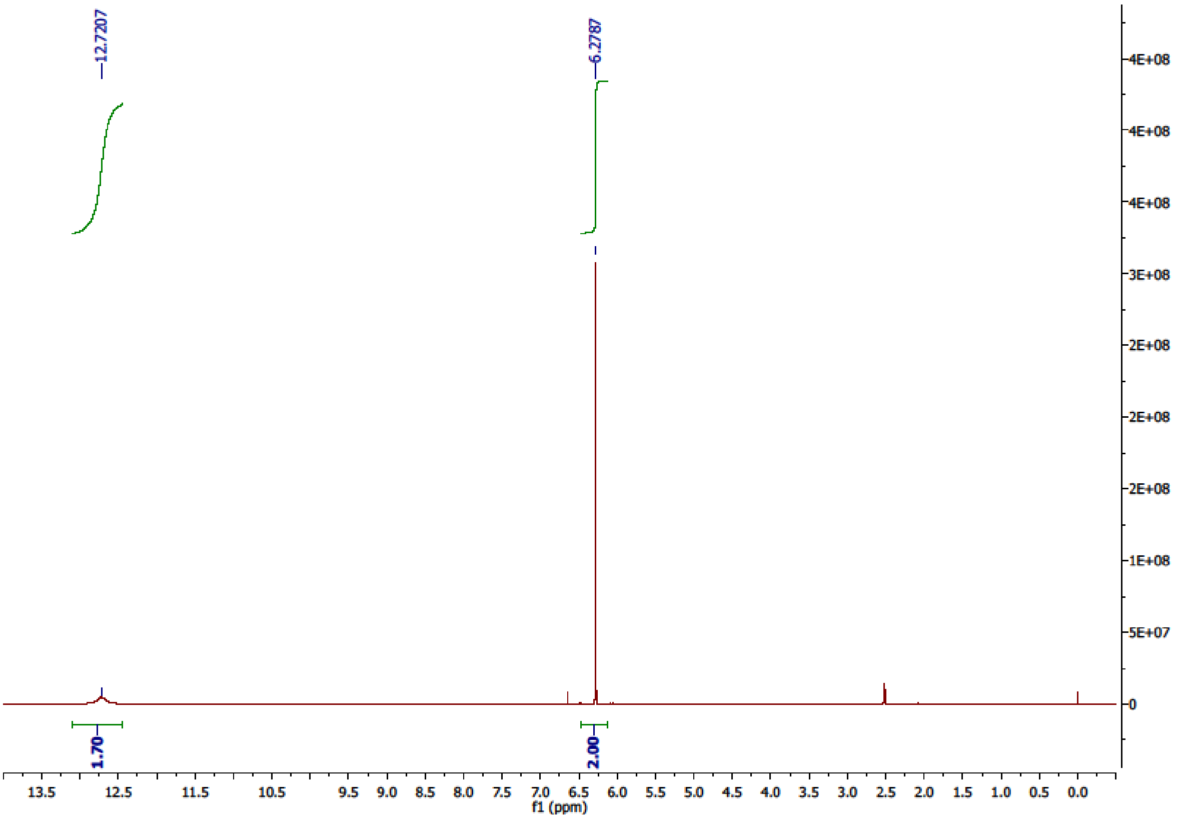

Figure S3. 1H[1] NMR spectra of maleic acid.
